# A Case of Third Dentition

**Published:** 1853-04

**Authors:** J. W. Keyes

**Affiliations:** Florida


*A case of third Dentition*. By J. W. KEYES, M. D., D. D. S. of Florida.

MR. EDITOR :—Through the Register, I wish to report a case of third dentition occurring in the person of Major Isaac Nathans, of this place.

The Major was of very slender frame and delicate constitution, until he reached his sixteenth year, but devoting much of his life to the erection of bridges, built up, under the vicissitudes to which his profession exposed him, a robust frame and iron constitution. Has been temperate in eating, and the use of ardent spirits. He is now in his sixty-ninth year, and as active and vivacious as a youth. In 1834, he had a violent and protracted attack of fever, was severely salivated, from which time until 1849, his lower teeth gradually dropped out in a sound condition.

In about a year after the loss of his lower incissors, his gums swelled, producing great pain and considerable fever, with constitutional disturbance.

He was confined to his bed, and after several days of suffering, a small, badly formed, ugly little central incissor made its appearance, and not long thereafter, another was thrust into light. They seem to have but little if any connection with the maxilla, and are of but little use. There are, at present, no symptoms that others will appear.

